# Functional Interaction among lncRNA HOTAIR and MicroRNAs in Cancer and Other Human Diseases

**DOI:** 10.3390/cancers13030570

**Published:** 2021-02-02

**Authors:** Monica Cantile, Maurizio Di Bonito, Maura Tracey De Bellis, Gerardo Botti

**Affiliations:** 1 Pathology Unit, Istituto Nazionale Tumori-Irccs-Fondazione G.Pascale, 80131 Naples, Italy; m.dibonito@istitutotumori.na.it; 2 Scientific Direction, Istituto Nazionale Tumori-Irccs-Fondazione G.Pascale, 80131 Naples, Italy; maura.traceydebellis@istitutotumori.na.it (M.T.D.B.); g.botti@istitutotumori.na.it (G.B.)

**Keywords:** lncRNA HOTAIR, miRNAs, cancer progression

## Abstract

**Simple Summary:**

This review aimed to describe the contribution of functional interaction between the lncRNA HOTAIR and microRNAs in human diseases, including cancer. HOTAIR/miRNAs complexes interfere with different cellular processes during carcinogenesis, mainly deregulating a series of oncogenic signaling pathways. A great number of ncRNAs-related databases have been established, supported by bioinformatics technologies, to identify the ncRNA-mediated sponge regulatory network. These approaches need experimental validation through cells and animal models studies. The optimization of systems to interfere with HOTAIR/miRNAs interplay could represent a new tool for the definition of diagnostic therapeutics in cancer patients.

**Abstract:**

LncRNAs are a class of non-coding RNAs mostly involved in regulation of cancer initiation, metastatic progression, and drug resistance, through participation in post-transcription regulatory processes by interacting with different miRNAs. LncRNAs are able to compete with endogenous RNAs by binding and sequestering miRNAs and thereby regulating the expression of their target genes, often represented by oncogenes. The lncRNA HOX transcript antisense RNA (HOTAIR) represents a diagnostic, prognostic, and predictive biomarker in many human cancers, and its functional interaction with miRNAs has been described as crucial in the modulation of different cellular processes during cancer development. The aim of this review is to highlight the relation between lncRNA HOTAIR and different microRNAs in human diseases, discussing the contribution of these functional interactions, especially in cancer development and progression.

## 1. Introduction

Long non-coding RNAs (lncRNAs) belong to the large family of ncRNAs, and are involved in many biological processes, such as cell migration, proliferation, cell cycle, apoptosis, and autophagy [1,2,3], and in numerous human diseases [4,5,6,7]. Many studies have also found that they play a main role in tumor initiation promotion and progression, and in drug resistance mechanisms [8,9,10,11].

LncRNAs can be divided into different classes in relation to genomic site, subcellular location (nuclear and cytoplasmic lncRNAs), and activity [12,13]. Most nuclear lncRNAs act by inducing transcriptional repression by guiding chromatin modifiers to specific genomic loci and recruiting DNA methyltransferases and histone modifiers, such as the Polycomb repressive complex PRC2 [14,15]. Other nuclear lncRNAs are able to function as decoys sequestering transcription factors [16]. Cytoplasmic lncRNAs are mainly involved in gene regulation mechanisms through different mechanisms: (i) some lncRNAs recognize complementary sequences of transcripts that can also co-localize in the same chromosomal region, and this interaction modulates their translation; (ii) other lncRNAS are able to modulate mRNA stability [17]; and (iii) most lncRNAs possess miRNA recognition elements (MREs), suggesting that lncRNAs are directly involved in synthesis, maturation, and degradation of miRNAs [18]. In theory, these lncRNAs act as competing endogenous RNAs (ceRNAs) by binding and sequestering specific miRNAs (‘miRNA sponges’) and further regulate mRNAs expression, preventing the repression of target mRNAs [19]. Consequently, lncRNA–miRNA physical interaction leads to a complex regulation network controlling gene expression at transcriptional, post-transcriptional, and post-translational levels. For this reason, a number of studies have highlighted that these interaction networks can be actively involved in many cell processes and in human diseases, including tumorigenesis [20,21].

The lncRNA HOX transcript antisense RNA (HOTAIR) has a crucial role in the initiation and progression of numerous human cancers, becoming a key diagnostic and predictive biomarker. Its aberrant activity can occur at different levels, as a molecular scaffold regulating chromatin status and transcriptional silencing but also via directly sponging different miRNAs to modulate the downstream gene expression [22]. Many studies reported functional interactions between *HOTAIR* and microRNAs able to modulate crucial cellular processes during cancer development [23,24].

In this review, we discuss the relation between lncRNA HOTAIR and different microRNAs, highlighting in particular the contribution of these functional interactions to tumor initiation and progression.

## 2. HOTAIR and Its Role in Cancer

HOTAIR is an lncRNA, 2158 bp long, located on chromosome 12q13.13 between *HOXC11* and *HOXC12* genes [25] and acts as a crucial modulator of chromatin re-modeling and transcriptional silencing [26]. *HOTAIR* is able to bind two different chromatin modifiers: (i) the *PRC2* (Polycomb repressive complex) at the 5′ end [25], which leads to the trimethylation of the H3K27 histone complex and subsequent transcriptional repression of differentiation genes [26]; and (ii) the lysine-specific histone demethylase 1A (*LSD1*) at the 3′ end [27]. HOTAIR functions as a molecular scaffold for the conjunction of the two complexes, leading to epigenetic changes contributing to gene silencing. For instance, the HOTAIR-PRC2-LSD1 complex can be redirected into a specific area in chromosome 2 where metastatic suppression genes are silenced [25,26,27,28].

The regulation of HOTAIR expression, both in silencing and in induction of its aberrant expression, is controlled by different molecular pathways [29].

It is well known that HOTAIR is transcriptionally induced by the estrogen hormone (17β-estradiol, E2) through multiple functional estrogen response elements (EREs) in the promoter region [30]. The induction of HOTAIR expression can often be mediated by the tumor microenvironment (TME). Different extracellular matrix (ECM) proteins, such as laminin, collagen I, and osteopontin, are able to induce HOTAIR overexpression, modulating PI3K/AKT pathways and promoting cell mobility and invasion [31]. TGF-β1 can induce *HOTAIR* expression, promoting epithelial mesenchymal transition (EMT) [32]. Moreover, c-Myc, recognizing a putative E-box element in the upstream region of *HOTAIR*, increases its expression and its promoter activity [33]. More recently, Liang et al. showed that miR-146a-5p is a potential upstream activator of HOTAIR [34].

Regarding HOTAIR suppression modulation, different epigenetic mechanisms, mediated by miRNAs, have been described in the literature. MiR-141 is able to bind to HOTAIR in a sequence-specific manner and suppresses its expression and functions [35]. Likewise, miR-203 inhibits HOTAIR modulating EMT [36]. MiR-101-3p, by binding and suppressing the expression of the serum response factor (SRF) gene, a HOTAIR transcription factor, can indirectly negatively regulate its transcription [37].

Although numerous studies have highlighted the crucial role of HOTAIR in several physiological cellular processes, indicating it as a cell cycle-related gene [38], most experimental observations are oriented to understanding its role in tumorigenesis [29]. In fact, the overexpression of *HOTAIR* has been related to the evolution of some solid cancers [26,39,40] with a crucial role in cancer initiation, progression, and drug sensibility modulation [41,42].

HOTAIR can interfere with the main molecular pathways related to breast cancer (BC) development. The HOTAIR promoter has several estrogen response elements (EREs) and estradiol agonists, which are able to induce its expression in in vitro and in vivo BC models [43]. *HOTAIR* is mainly involved in the definition of metastatic risk in BC molecular subtypes [44,45,46]. HOTAIR is also a critical modulator of autophagy [47], epithelial mesenchymal transition (EMT) [32], and drug resistance in BC [42].

In non-small cell lung cancer (NSCLC) patients, HOTAIR overexpression is mainly associated with cisplatin resistance [48]. In prostate cancer, HOTAIR upregulation promotes cell growth and invasion by blocking the degradation of androgen receptor (*AR*) protein to which it binds [49]. In bladder cancer, HOTAIR is a prognostic biomarker involved in chemo sensitivity to doxorubicin [50,51]. Aberrant HOTAIR expression has also been described in ovarian and cervical cancer, with a powerful association with metastatic progression and poor patient survival [52,53]. In endometrial carcinoma, HOTAIR overexpression has a prognostic value and is associated with cisplatin resistance [54]. HOTAIR upregulation appears closely associated with clinic-pathological characteristics and tumor progression in colorectal cancer [55] and gastric cancer [56]. In laryngeal squamous cell carcinoma, aberrant HOTAIR expression correlates with stages, metastases, poor prognosis, and the modulation of cisplatin sensitivity [57].

In most human cancers, HOTAIR shows an important prognostic and predictive role as a circulating biomarker, especially in BC [42], lung cancer [58], cervical cancer [59], liver cancer [60], and lastly in gastric cancer patients [56].

Many other studies also described the contribution of HOTAIR to tumor microenvironment (TME) intracellular signaling, especially to tumor phenotype modifications during metastatic evolution [31]. HOTAIR silencing in cancer cells is able to inhibit EMT by inducing E-cadherin expression and vimentin and beta-catenin repression [32]. *HOTAIR* is a crucial modulator of cancer stem cells (CSCs), able to induce proliferation, colony formation, migration, and self-renewal capacity. Moreover, HOTAIR has a pro-angiogenic activity [61] and it promotes TAM and MDSCs proliferation [62]. Aberrant *HOTAIR* expression has been described in lymphocytes surrounding the metastatic tumor cells of metastatic melanoma patients [31,63].

## 3. HOTAIR and MiRNAs Interaction in Embryonic Development and Human Diseases

LncRNAs play a crucial role in cell differentiation and embryonic development [64,65,66,67,68].

Several studies highlighted the role of HOTAIR in normal mammal development [69]. *HOTAIR* is not expressed in the early stage of zygotes [70] yet its activity is associated with the early stages of embryogenesis [71]. It contributes to the development of the lumbosacral region [25] skin and uro-genital system due to its ability to modulate the expression of some genes of the HOX gene network [72]. HOTAIR can also be involved in osteogenesis, modulating the expression of two osteogenic-related genes, *ALPL* and *BMP2* [73].

The functional interaction between lncRNA and miRNAs is fundamental during embryonic development in mammals [74,75,76,77]. The ability of HOTAIR to function as a competitive endogenous RNAs (ceRNA) to regulate gene expression competing with microRNA binding sites is often the basis of the processes related to cell differentiation and development. MiRNA-130b-3p is a direct target of HOTAIR and its expression is negatively regulated by HOTAIR during vasculogenesis. HOTAIR can influence the proliferative and apoptotic abilities of vascular smooth muscle cells (VSMCs) by regulating the microRNA-130b-3p/peroxisome proliferator-activated receptor alpha (PPARα) axis [78]. Autophagy can lead to cellular changes necessary for proper differentiation and/or development in mammals, especially for preimplantation development, survival during neonatal starvation, and cell differentiation during erythropoiesis, lymphopoeisis, and adipogenesis [79]. HOTAIR is able to upregulate the expression of autophagy-related 3 (ATG3) and autophagy-related 7 (ATG7), sponging-specific miRNAs involved in the suppression of these two genes [80].

The de-regulation of many lncRNAs and their functional relationship with miRNAs has also been described during the pathogenesis of different human diseases [81,82].

HOTAIR is involved in osteogenic differentiation by regulating osteogenic differentiation markers RUNX2, COL1A1, and ALP. This process is carried out through modulation of the expression of miR-17-5p and its target gene SMAD7. Further, the HOTAIR/miR-17-5p axis is responsible for the progression of non-traumatic osteonecrosis of the femoral head (ONFH) [83]. Likewise, HOTAIR overexpression plays an important role in osteoarthritis progression, correlating with the modified Rankin scale, extracellular matrix (ECM) degradation, and chondrocytes apoptosis. The same HOTAIR/miR-17-5p axis contributes to the progression of this disease via the wnt/β-catenin pathway. HOTAIR has been described as a regulator of inflammatory injury, cell apoptosis, and influx of inflammatory cytokines during the progression of osteoarthritis. It performs its function by downregulating miR-17-3p, which in turn is negatively regulated by ETV1 expression by activation of the MAPK/c-Jun and NF-κB pathway [84]. HOTAIR is de-regulated in intervertebral disc degeneration (IDD) tissues [85]. In these cells, it acts as a microRNA-34a-5p sponge, which sequesters miR-34a-5p, and reduces nucleus pulposus cell apoptosis, increasing Notch1 expression. This process can be directly involved in IDD progression when activated [86]. Moreover, HOTAIR modulates the expression of α-SMA, a key cellular element of tissue fibrosis. The increase in the number of myofibroblasts in vivo correlates with the disease severity of systemic sclerosis (SSc) and they display high levels of HOTAIR. Overexpression of HOTAIR in dermal fibroblasts induced an EZH2-dependent increase in collagen and α-SMA expression in vitro, as well as repression of miRNA-34A expression and consequent NOTCH pathway activation [87]. HOTAIR upregulation also correlates with the progression of liver fibrosis. Its knockdown resulted in a reduction in type I collagen and α-SMA and increased PTEN expression. In liver tissues and cells, HOTAIR downregulates miR-29b expression and attenuates its control on epigenetic regulation, leading to enhanced PTEN methylation and contributing to the progression of liver fibrosis [88].

A lot of studies showed the crucial role of HOTAIR in Parkinson’s disease (PD) [89], highlighting its interaction with different miRNAs. HOTAIR shows high expression levels in PD cells and in the PD mice models, acting as a ceRNA for miR-126-5p to modulate *RAB3IP* expression during PD development [90]. In human neuroblastoma cells exposed to different concentrations of 1-methyl-4-phenylpyridinium (MPP^+^), a molecule able to induce neuronal injury, mimicking PD in vitro models [91], HOTAIR is surprisingly increased in a dose-dependent manner. HOTAIR in these cells acts as a sponge for miR-874-5p, inducing its downregulation and thereby modulating the autophagy-related 10 (ATG10) gene [92]. Furthermore, HOTAIR positively regulates the ELAVL1 gene, a modulator of inflammatory and angiogenic processes, by targeting miR-326. HOTAIR silencing significantly inhibits neuronal damage through repression of the NLRP3 gene via regulation of the miR-326/ELAVL1 axis in PD [93].

HOTAIR is aberrantly expressed and inhibits the proliferation, migration, and invasion of trophoblast cells, in preeclampsia placental tissues and cell lines. Its role during preeclampsia is related to targeting of miR-106a. HOTAIR is able to modulate miR-106a transcription and trophoblast cell migration and invasion in an EZH2-dependent manner [94].

IL-13 overexpression is responsible for the development of neonatal bronchial hyper-responsiveness (BHR). HOTAIR can modulate the expression of IL-13 during BHR progression through negative regulation of miR-126.

The functional interaction between HOTAIR and miR-126 has also been described in cardiac dysfunction. HOTAIR is able to aggravate myocardial ischemia-reperfusion by competitively binding serine/arginine splicing factor 1 (SRSF1) with microRNA-126. The HOTAIR/microRNA-126/SRSF1 axis can be responsible for influencing the development of myocardial dysfunction [95]. Many other studies showed that HOTAIR plays a key role in the development of cardiovascular diseases [96,97,98,99,100], highlighting specifically a cardioprotective effect partly associated with HOTAIR-miR-125 or HOTAIR-miR-1 interactions [99]. In addition, the interaction between HOTAIR and miR-519d-3p, responsible for downregulation, alleviated myocardial infarction or hypoxia-induced cardiomyocyte apoptosis in animal models [100]. HOTAIR is also involved in diabetic cardiomyopathy. Its overexpression in cardiac cells protects against diabetes-related cardiac dysfunction, oxidative damage, inflammation, and cell death in vivo. The protective effect of HOTAIR has been mediated by direct interaction with miR-34a and indirect modulation of SIRT1, a target of miR-34a [101,102].

HOTAIR directly targets miR-138 and is associated with the development of rheumatoid arthritis, because it is involved in maintaining the chondrocyte phenotype. HOTAIR could target miR-138 to suppress its expression, and this process had a reversed correlation in LPS-induced chondrocytes. Therefore, HOTAIR could play a protective role in rheumatoid arthritis through the regulation of miR-138 expression and the NF-kB signaling pathway.

## 4. HOTAIR and MicroRNAs Interaction in Cancer

Numerous studies have highlighted the aberrant cellular activity produced by the interaction of HOTAIR with different miRNAs during cancer development and progression (Table 1).

### 4.1. Breast Cancer

De-regulation of HOTAIR has been mostly described in the evolution of breast cancer (BC) [39]. Gupta et al. described overexpression of HOTAIR in primary BC tumors with high metastatic potential and poor survival rate, suggesting its predictive role in BC tumor progression [44]. HOTAIR is considered a metastatic BC biomarker, especially in basal-like BCs [145]. In triple-negative breast cancer (TNBC) patients, high *HOTAIR* expression in tumor tissues is strongly correlated with lymph node metastasis, and it is directly associated with the androgen receptor (AR) [46].

In breast cancer cells, miR-7 overexpression inhibits cell migration and invasion, partially reversing EMT by targeting *SETDB1*. It is able to interact with HOTAIR and this functional interaction reduces the expression of miR-7, inhibits HoxD10 expression, and promotes the invasiveness and metastasis of BC cells [105].

HOTAIR overexpression is regulated by estradiol through GPER via BC cells while miR-148a is able to significantly suppress HOTAIR expression in the same cells. The complex regulation mechanism of HOTAIR, by estradiol, could be directly associated with HOTAIR/miR148a functional interaction [116]. HOTAIR silencing in BC cells increases cellular sensitivity to ionizing radiation, promoting DNA damage and cell cycle arrest. HOTAIR regulates cellular radiosensitivity through suppression of miR-218, suggesting that HOTAIR-miR-218 signaling is deeply involved in the mechanism of radiosensitization [131]. Additionally, HOTAIR is able to modulate the radiosensitivity mechanism through post-transcriptional upregulation in the stress-inducible oncogene HSPA1A. HOTAIR facilitates expression of the HSPA1A by sequestering miR-449b-5P, its direct suppressor. The HOTAIR/miR-449b-5p/HSPA1A axis represents a new mechanism associated in vitro and in vivo with BC radioresistance [138].

Delphinidin, an anthocyanidin with potent anticancer properties, modulates BC cell proliferation and migration via negative regulation of HOTAIR. In breast and tumor tissues, delphinidin treatment also inhibits the expression of β-catenin, p-GSK-3β, c-Myc, cyclin-D1, and MMP-7 and upregulates miR-34a, a master regulator of tumor suppression. Overexpression of HOTAIR is responsible for blocking the effect of delphinidin on miR-34a in BC cells [146].

MiR-146a-5p has been described as a potential upstream activator of HOTAIR in TNBC cells and their functional interaction is able to promote migration and invasion of cancer cells with a prognostic role in TNBC patients [34]. Moreover, HOTAIR can inhibit vasculogenic mimicry and cell migration in TNBC cells by sequestering miR-204, a tumor suppressor microRNA, frequently downregulated in BC, involved in angiogenesis, cell migration, as well as in the modulation of vasculogenic mimicry. HOTAIR contains a conserved potential binding site for miR-204 at the 131–145 nucleotide position, and silencing of HOTAIR in TNBC cells strongly increases miR-204 expression levels [124].

### 4.2. Glioma

HOTAIR has a prognostic role in glioma patients, as a critical regulator of cell cycle progression [147,148]. HOTAIR silencing in glioma cells leads to upregulation of miR-326 expression and it is able to suppress tumor growth and prolong the survival of nude mice, downregulating FGF1, p-PI3K/PI3K, p-AKT/AKT, and p-MEK1/2/MEK1/2 expression [133].

Another tumor suppressor miRNA involved in HOTAIR regulation during glioma progression is miR-14, frequently downregulated in glioma tissue samples and cells. In glioma tissues, an inverse correlation between miR-141 and HOTAIR expression has been detected. MiR-141 can interact with HOTAIR by combining directly with the 3′UTR of HOTAIR in cancer cells, also modulating the expression of the spindle and kinetochore-associated complex subunit 2(SKA2) gene, involved in the maintenance of the metaphase plate and/or spindle checkpoint silencing [114]. Additionally, miR-148b-3p is able to bind HOTAIR, in a sequence-specific manner, suppressing its expression and thereby reducing proliferation, cell cycle progression, and invasion of glioma cells [118]. Similarly, miR-125a-5p is also able to downregulate HOTAIR modulation, through mTOR expression, proliferation, and migration of glioma cells [149].

### 4.3. Lung Cancer

HOTAIR is able to interact with miR-326, negatively modulating its expression. MiR-326 acts as a tumor suppressor gene. It is able to target Phox2a, regulating proliferation, migration, cell cycle, and apoptosis in lung cancer cells and inhibiting tumor growth in nude mice. HOTAIR silencing increases the accumulation of miR-326 in lung cancer cells [134]. Further, miR-326 overexpression is able to reverse cisplatin chemoresistance of lung cancer cells in vitro and in vivo and this activity is strongly related to aberrant HOTAIR expression [135].

HOTAIR is upregulated in NSCLC cell lines and its downregulation significantly represses cell proliferation and inhibits cell migration and invasion by facilitating miR-217 expression. Bioinformatics analysis and Luciferase reporter assay identified DACH1 as a direct target of miR-217, suggesting that the HOTAIR/miR-217/DACH1 signaling pathway is strongly involved in lung cancer progression [128].

### 4.4. Urinary Tract Tumors

The functional interaction between HOTAIR and miRNAs have largely been described in uro-genital tumors. In bladder cancer cells and tissues, HOTAIR, via the recruitment of PRC2 and LSD1, is directly involved in the regulation of miR-205 expression. This functional interaction blocks the expression of miR-205, and promotes migration and invasion of bladder cancer cells [24]. In renal cancer cells, HOTAIR is able to interact with miR-141 in a sequence-specific manner. This interaction suppresses *HOTAIR* expression in an Ago2 (Argonaute2)-dependent manner, and reduces the proliferation and invasion ability of cancer cells [35]. HOTAIR expression is negatively correlated with miR-217 in human renal cancer tissues and cells, in this way modulating proliferation and migration, and inhibiting apoptosis. Additionally, HOTAIR functions as a ceRNA for miR-217 to induce HIF-1α/AXL signaling, facilitating renal cancer progression both in vitro and in vivo. Furthermore, in renal cancer cells, ERβ could modulate HOTAIR, and consequently antagonize, in addition to miR-217, other miRNAs (miR-200c, miR-138, miR-204) to upregulate specific oncogenes, such as metalloproteinase domain 9 (ADAM9), cyclin D2 (CCND2), EZH2, VEGFA, vimentin (VIM), and zinc finger E-box binding homeobox 1 and 2 (ZEB1 and ZEB2), to promote RCC proliferation and invasion [125].

Several studies have described the functional interaction between HOTAIR and miRNAs in prostate cancer development and progression. HOTAIR is upregulated by genistein in castration-resistant prostate cancer cell lines and its knockdown decreases cell proliferation, migration, and invasion, and induces apoptosis and cell cycle arrest. The tumor suppressor miR-34a is also upregulated by genistein, directly targeted HOTAIR, modulating cell proliferation, migration, and invasion, in both PC3 and DU145 PCa cells [107]. In prostate cancer cells, EZH2 interacts with HOTAIR to silence miR-193a, a tumor suppressor miRNA, through the introduction of tri-methylation of H3K27 at the miR-193a promoter region. On the other hand, miR-193a directly targets HOTAIR and reduces its expression in prostate cancer cells [121]. Furthermore, HOTAIR is negatively correlated with the expression of miR-152 during prostate cancer evolution [119].

### 4.5. Gynecological Cancers

The upregulation of HOTAIR contributes to the progression of ovarian cancer acting as a ceRNA to regulate the expression of Rab22a through the competitive binding of miR-373. Rab22a is a small GTPase involved in cancer cell proliferation through the integrin-mediated signaling pathway [150]. In ovarian cancer cells, HOTAIR positively stimulates *CCND1* and *CCND2* genes, whose upregulation is associated with tumor progression [126]. This activity is related to the negative modulation of miR-206 expression, and the ceRNA regulatory network *HOTAIR-miR-206-CCND1/CCND2* contributes to the promotion of ovarian cancer cell proliferation, cell cycle progression, migration, and invasion [126].

MiR-200c overexpression in ovarian cancer cells and animal models is able to downregulate *HOTAIR* expression and simultaneously decrease Snail and increase E-cadherin expression, significantly reducing the invasion and tumorigenicity of cancer cells. These data suggest that the functional interaction between miR-200c and *HOTAIR* plays a crucial role during ovarian cancer progression through the modulation of EMT [123]. Furthermore, the downregulation of HOTAIR is able to reverse cisplatin resistance of ovarian cancer cells by upregulation of miR-138-5p. It, in turn, can directly regulate the expression of EZH2 and SIRT1, which have been proven to be involved in the regulation of cisplatin resistance in ovarian cancer [151]. In addition, other miRNAs (such as miR-34a and miR-454) can be involved in regulating the chemosensitivity of ovarian cancer through the functional interaction with HOTAIR [113]. HOTAIR plays an important oncogenic role in cervical cancer, promoting the proliferation, migration, and invasion of cancer cells. In cervical cancer cells, HOTAIR also promotes the expression of the human leukocyte antigen-G (HLA-G), a newly identified member of the non-classical MHC family, through inhibition of miR-148a expression. The aberrant activity of the HOTAIR-miR-148a-HLG-A axis could represent a new oncogenic mechanism in cervical cancer pathogenesis. Moreover, miR-17-5p expression is inversely correlated with HOTAIR and it is able to directly target HOTAIR 3′-UTR. HOTAIR via sponging miR-17-5p can promote proliferation, migration, and invasion of cervical cancer cells [106].

### 4.6. Gastric Cancer

Many studies have highlighted the functional interaction between HOTAIR and different miRNAs in gastric cancer (GC). The crucial prognostic role of HOTAIR in this tumor type is well documented and in addition to being aberrantly expressed it is also directly related to the clinical-pathological characteristics and poor survival of patients [152]. In GC cells, miR-331-3p and miR-124 can directly bind to HOTAIR through respective miRNA recognition sites. Moreover, HOTAIR is able to modulate the expression of the miR-331-3p target, HER2. The expression of HER2 is positively associated with upregulated HOTAIR in GC tissue samples, suggesting that the activation of the miR-331-3p/HER2/HOTAIR axis might be biologically significant in human gastric tumorigenesis [137]. Some miRNAs interact with HOTAIR to promote GC progression: (i) miR-101-3p, described as a tumor suppressor in GC, inhibits HOTAIR expression and promotes the proliferation and invasion of GC cells through direct targeting of SRF; (ii) miR-217 expression is negatively regulated by interaction with HOTAIR and thereby increases the expression of GPC-5, a target of miR-217 in GC, promoting GC development; and miR-152 is negatively regulated by the direct interaction with HOTAIR. This interaction is also involved in the modulation of HLA-G expression. HOTAIR acts as a ceRNA to regulate HLA-G expression by sponging miR-152 during GC progression [120]; and miR-454-3p is able to interact with HOTAIR, modulating its expression and influencing GC cells’ growth via the STAT3/cyclin D1 pathway [139]. HOTAIR overexpression is strongly associated not only with migration, invasion, and metastasis, but also with the EMT mechanism in gastric cancer both in vitro and in vivo. This process is achieved by binding of HOTAIR to PRC2, which epigenetically silences miR34a and simultaneously activates Snail transcription [153].

### 4.7. Colon Cancer

Mirna 34a can interact with HOTAIR as well as in colon cancer cells (CRCs). HOTAIR is able to modulate miR-34a, inhibiting its expression, and thereby promoting cell migration and metastasis of CRC cells [154]. MiR-545 negatively regulates cell proliferation and inhibits EGFR expression by affecting its 3′-UTR activity. Its expression is suppressed by HOTAIR overexpression whereas it is enhanced by HOTAIR silencing, suggesting that the HOTAIR-microR-545-EGFR axis is fundamental in regulating CRC cell proliferation [141]. MiR-326 expression is also downregulated in CRC patients with distant metastasis and in CRC cells. HOTAIR acts as a molecular sponge for miR-326 and negatively regulates FUT6 expression, a direct target of miR-326 involved in the modulation of the PI3K/Akt pathway in human cancer [155]. Mechanically, HOTAIR–miR-326 interaction mediates α1, 3-fucosylation biosynthesis on the HCELL glycoform of CD44. Consequently, the HOTAIR/miR-326/FUT6/CD44 axis is able to modulate the activity of the PI3K/AKT/mTOR pathway during CRC progression [136]. Another crosstalk involving HOTAIR and miR-214 has been described in CRC development. HOTAIR acts as a sponge for miR-214, regulating *ST6GAL1* expression. The latter induces elevated metabolic sialylation of c-Met, which is co-mediated by HOTAIR and miR-214. In turn, sialylated c-Met affects the activity of the JAK2/STAT3 signaling pathway, suggesting that the HOTAIR/miR-214/*ST6GAL1* axis is crucial in CRC progression [127].

### 4.8. Liver Cancer

HOTAIR negatively regulates miR-218 expression in hepatocellular carcinoma (HCC) cells through an EZH2-miR-218-2 promoter regulatory axis, and this functional interaction increases cell growth and proliferation. In fact, mechanistically, miR-218 inhibits cell growth and induces G1-phase arrest by directly suppressing Bmi-1 expression, thereby activating the P14^ARF^-P53 and P16^Ink4a^-Rb signaling pathways in HCC [132]. Several other functional interactions have been described in HCC. FOXC1 binding to the upstream region of HOTAIR, induces its overexpression, and in turn, HOTAIR is able to negatively regulate the expression of miRga-1, promoting HCC cell proliferation and progression in tumor xenografts [103]. HOTAIR negatively regulates miR-122 expression in HCC cells. Its silencing is able to inhibit tumorigenicity both in vitro and in vivo by upregulating miR-122 expression. On the contrary, downregulation of miR-122 induced by HOTAIR may directly reactivate cyclin G1 expression, promoting growth and proliferation of HCC cells [111]. Similarly, miR-217-5p, described as a suppressor of cell progression by regulating the JAK3/STAT3 signaling pathway [156], is negatively regulated by HOTAIR. This functional interaction promotes cell proliferation, migration, invasion, and EMT in HCC cells. HOTAIR is also able to inhibit the expression of miR-145 by competitively recruiting PRC2 in HCC cells. The HOTAIR/miR-145 axis leads to TGF-β1 upregulation and promotes HCC cell progression [115].

### 4.9. Pancreatic Cancer

HOTAIR directly regulates miR-34a expression, binding to its promoter, and it is able to repress miR-34a expression through EZH2 mechanisms in pancreatic cancer cells [157]. In pancreatic cancer, HOTAIR acts as a miRNA sponge for other miRNAs to modulate different crucial pathways involved in tumor progression. MiR-663b has been described as a tumor suppressor in pancreatic cancer cells, able to repress IGF2 expression via targeting of its 3’UTR, thereby suppressing cell proliferation. In vitro mechanistic studies have revealed that miR-663b activity can be blocked by its interaction with HOTAIR, suggesting that miR-663b is epigenetically repressed by HOTAIR and exerts its tumor suppressive function via targeting of IGF2 in pancreatic cancer [144]. In addition, the same authors also highlighted that miR-613 has a suppressive role in pancreatic cancer, via targeting of neurogenic locus notch homolog protein 3 (notch3), and that HOTAIR functions as a competing endogenous RNA to regulate notch3 expression by sponging miR-613 [142].

### 4.10. Esophageal Cancer

In esophageal squamous cell carcinoma (ESCC), HOTAIR acts as a miR-148a sponge to positively regulate Snail2 expression, enhancing cell invasion and metastasis, and thereby promoting EMT [117]. Furthermore, HOTAIR is able to promote tumor invasiveness and progression in ESCC cells by acting as a miR-130a-5p sponge. This functional interaction leads to tumor progression via targeting of ZEB1, a transcription factor that promotes tumor invasion and metastasis by inducing epithelial-mesenchymal transition (EMT). Additionally, HOTAIR expression is upregulated by CCL18 in ESCC cells, suggesting that the CCL18/HOTAIR/miR-130a-5p/ZEB1 axis is crucial in the modulation of malignant progression of ESCC [112].

### 4.11. Other Cancers

Many studies reported aberrant activity of HOTAIR during head and neck tumor development and progression [158]. HOTAIR acts as a sponge and negatively regulates the expression of miR-101, modulating tumor progression of nasopharyngeal carcinoma cells. In turn, miR-101 negatively regulates COX-2 expression by directly targeting 3′-UTR of COX-2 mRNA. This HOTAIR/miR-101/COX-2 regulatory axis is crucial in nasopharyngeal carcinoma development [110].

miR-206 inhibits the activation of the PI3K/AKT signaling pathway by targeting STC2 in HNSCC cell lines, reducing cell growth and proliferation. HOTAIR is able to interact, with miR-206 silencing it and consequently promoting HNSCC cell proliferation, invasion, and migration [159].

In sarcoma cells, although the role of HOTAIR is well documented [160], there are scarce data about its role as an miRNAs sponge. HOTAIR mediates cell growth by negatively regulating miR-454-3p expression in chondrosarcoma cells, through an EZH2-mediated mechanism. HOTAIR knockdown results in growth inhibition of chondrosarcoma cells via miR-454-3p upregulation and Stat3 signaling inactivation in vivo [140].

During osteosarcoma progression, HOTAIR may act as a sponge for miR-217, involved in the repression of growth, migration, and invasion of osteosarcoma cells. This interaction decreases miR-217 and increases ZEB1 gene expression, a direct target of miR-217 able to induce tumor progression, promoting EMT with the activation of stem cell traits, immune evasion, and epigenetic reprogramming [129].

HOTAIR acts as a sponge for miR-613, downregulating its expression in retinoblastoma. This functional interaction, upregulating c-met expression, promotes viability, proliferation, apoptosis, and the EMT process of human retinoblastoma cells [143]. Two recent studies described the ability of HOTAIR to bind to different miRNAs during medulloblastoma progression. Zhang et al. showed that HOTAIR negatively regulates miR-1 and miR-206 expression in medulloblastoma cells. These miRNAs directly target YY1, a transcription factor involved in cell survival, proliferation, and EMT modulation. HOTAIR, downregulating miR-1 and miR-206, increases YY1 expression, promoting tumor growth, migration, invasion, and EMT of medulloblastoma cells [104]. Another molecular axis involving HOTAIR, miR-483-3p, and CDK4 is strongly associated with medulloblastoma progression. HOTAIR acts as a sponge for miR-483-3p, decreasing its expression and thereby upregulating CDK4, a direct target of miR-483-3p. On the contrary, HOTAIR silencing inhibits cell proliferation and promotes cell apoptosis in medulloblastoma cells by upregulation of miR-483-3p and downregulation of CDK4, respectively.

Nevertheless, the main indications of functional interactions between HOTAIR and miRNAS come from studies carried out on solid tumors, and a series of data also report their crucial role in hematological tumors. HOTAIR plays an important oncogenic role in acute myeloid leukemia (AML) (REF). In AML blasts, HOTAIR affects the expression of endogenous miR-193a target, a tumor suppressor gene in AML, able to represses c-kit expression [161]. HOTAIR/miR-193a interaction, upregulating c-kit, promotes malignant evolution of AML blasts. The role of the HOTAIR/miR-193a/c-Kit regulatory pathway has been validated in 70 patients with AML, showing a strong association with prognosis and disease outcome [122].

## 5. Prediction of lncRNA–miRNA Interactions

The interaction between miRNAs and lncRNAs is accepted to be important for gene regulation and consequently is involved in all processes related to normal cell physiology, and it also can contribute to the initiation and development of various types of complex diseases [162]. This complex network of molecular interactions is the basis of “network medicine”, which uses network science approaches to investigate disease pathogenesis [163]. Recently, a great number of ncRNAs-related databases have been established, supported by bioinformatics technologies. Most of them aim to identify the ncRNA-mediated sponge regulatory network, especially in tumor diseases. The Gene Expression Omnibus database repository (GEO) (https://www.ncbi.nlm.nih.gov/geo/), containing high-throughput gene expression data, and the Cancer Genome Atlas (TCGA) database (https://cancergenome.nih.gov/) are among the most used data mining tools. They have allowed prediction and integration of differentially expressed RNAs, producing many differentially expressed lncRNA-miRNA-mRNA ceRNA networks [164,165,166,167,168]. Many different bioinformatic tools were merged to predict ceRNA networks, such as miRcode MiRDB miRTarBase miRanda, Targetscan, GDCRNATools, and starBase v2.0 [169,170,171]. A broader overview of bioinformatics platforms and their applications are summarized in Table 2. These tools, by identifying RNA—RNA disease-related networks and allowing the definition of new potential diagnostic biomarkers and therapeutic targets, necessarily need experimental validation using approaches based on cells and animal models.

Since the network of interactions involving miRNAs and lncRNAs is vast and complex, and their identification is essential for providing a better understanding of the complex mechanisms in which they are involved, it was necessary to develop large-scale prediction models that exploit the advancement of deep learning. Several deep learning-based methods have been developed to predict lncRNA–miRNA interaction. Some examples are represented by: (i) the Expression Profile-based prediction model for LncRNA-MiRNA Interactions (EPLMI), the first technique developed for large-scale lncRNA-miRNA interaction profiling. This model analyzes the patterns in large-scale expression profiles of known lncRNA–miRNA interactions and it is based on the assumption that lncRNAs that are very similar to each other tend to have similar interaction or non-interaction patterns with miRNAs and vice versa [172]; (ii) the Graph Convolution for novel LncRNA-MiRNA Interactions (GCLMI), based on graph convolutional networks (GCNs), a powerful type of neural network designed to work directly on graphs and leverage their structural information. GCLMI is a network-based algorithm that considers known lncRNA–miRNA interactions along with lncRNA and miRNA expression levels, and aims to calculate the possibility that an lncRNA–miRNA pair is interactive in biological processes [173]; (iii) the Linear Neighbour Representation to predict LncRNA-MiRNA Interactions (LNRLMI) is based on the implementation of an lncRNA–miRNA network realized combining the known interaction network and similarities based on expression profiles of lncRNAs and miRNAs. To predict the new links of the known lncRNA–miRNA interaction network, a linear optimization, a semi-supervised model, has been added to this model [174].

Numerous studies have been carried out to predict potential HOTAIR miRNA targets. They used different online miRNA target prediction algorithms combined with each other (Table 2). For example, direct HOTAIR miRNA targets were identified with the TargetScan algorithm in prostate [175] and esophageal cancer cells [176]. In HCC tissues and cell lines, the prediction algorithms PicTar, miRanda, TargetScan, and Microcosm Targets were used. In gastric cancer cells, the potential microRNAs binding sites of HOTAIR have been predicted by the computer algorithm miRanda and by combined RegRNA and microRNA.org-target program [130].

## 6. Conclusions and Perspectives

LncRNAs and miRNAs are the most studied classes of non-coding RNAs involved in human diseases, including cancer. However, it is strongly emerging that the complex network of interactions in which they are involved, also defined as the ‘sponge effect’, may be even more important in understanding the molecular mechanisms involved in pathologic conditions. HOTAIR is a key regulator of chromatin status and a mediator of transcriptional silencing. Its function in cancer has been very well documented, highlighting in particular its role in the molecular mechanisms associated with metastatic progression and drug resistance. In pathological conditions, HOTAIR can have both nuclear localization, mainly associated with its activity of the molecular scaffold for the assembly of multiple-component complexes, such as ribonucleoprotein (RNP) complexes, and cytoplasmic localization, which allows it to target specific miRNA transcripts and modulate miRNA stability. Thus, the identification of its precise cellular sub-localization is fundamental in understanding its cellular activity, establishing the best system to inhibit it. The clinical relevance of HOTAIR as a biomarker in human cancer is well established. Beyond its enormous prognostic value, HOTAIR also represents a crucial circulating biomarker [63] with excellent potential as a therapeutic target [42]. Although only one clinical study has been registered in the *ClinicalTrials.gov* database (https://clinicaltrials.gov/ct2/show/results/NCT03469544), a series of compounds, specifically capable of blocking the activity of some lncRNAs, are already used in clinical practice. Recently, small molecules able to block HOTAIR activity by interfering with HOTAIR/EZH2 scaffold interaction have been validated in cell and murine tumor models [177,178].

**Figure 1:**
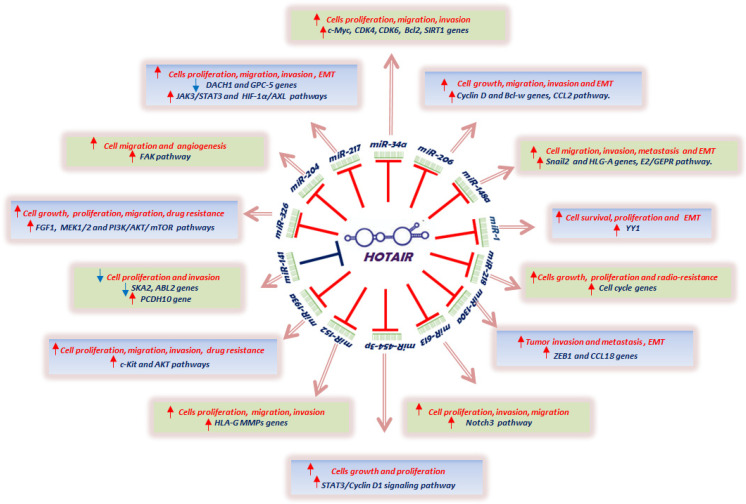
Schematic representation of principal functional interactions between lncRNA HOTAIR and different microRNAs. HOTAIR/miR-34a interaction promotes tumor cell proliferation, migration, and invasion by induction of the transcription factor c- Myc, the cyclin-dependent kinases CDK4 and CDK6, and anti-apoptotic proteins Bcl2 and SIRT1 in breast, gastric, pancreatic, prostate, and colon cancers; HOTAIR/miR-217 interaction modulates cell proliferation, migration, invasion, and epithelial mesenchymal transition (EMT) by downregulation of tumor suppressor genes DACH1 and GPC-5, and the activation of JAK3/STAT3 and HIF-1α/AXL signaling pathways in lung, gastric, and kidney cancers and in osteosarcoma; HOTAIR/miR-204 interaction induces cell migration and angiogenesis by the tyrosine kinase FAK signaling pathway in breast and renal cancers; HOTAIR/MiR326 interaction promotes tumor cell growth, proliferation, migration, and chemoresistance by induction of the FGF1, MEK1/2, and PI3K/AKT/mTOR signaling pathways in lung, colon, and glioma cancers; HOTAIR/Mir-141 interaction reduces tumor cell proliferation and invasion by decreasing the cell-cycle gene SKA2 and anti-apoptotic gene ABL2, and inducing the metastasis suppressor gene PCDH10 in renal cancer and glioma; HOTAIR/Mir139a interaction promotes tumor cell proliferation, migration, invasion, metastasis, and drug resistance by induction of the receptor tyrosine kinase c-Kit and AKT signaling pathways in prostate cancer and myeloid leukemia; HOTAIR/Mir152 interaction induces tumor cell proliferation, migration, and invasion by modulation of the human leukocyte antigen G (HLA-G) and metalloproteinases expression in prostate and gastric cancer; HOTAIR/Mir454-3p interaction modulates tumor cells growth and proliferation by induction of the STAT3/cyclin D1 signaling pathway in gastric cancer and chondrosarcoma; HOTAIR/Mir613 promotes cell proliferation, invasion, and migration by modulation of the cell surface receptor notch3 signaling pathway in pancreatic cancer and retinoblastoma; HOTAIR/Mir130a increases tumor invasion and metastasis by inducing EMT and upregulates the transcription factor ZEB1 and the chemokine ligand CCL18 in ESCC and gallbladder cancers; HOTAIR/Mir218 promotes tumor cell growth, proliferation, and resistance to ionizing radiation by induction of cell cycle genes in breast and liver cancers; HOTAIR/Mir1 interaction induces cell survival, proliferation, and EMT by upregulation of the cancer metastasis gene YY1 in liver cancer and medulloblastoma; HOTAIR/Mir148a interaction increases cancer cell migration, invasion, metastasis, and EMT by inducing the Snail2 gene, Estradiol (*E2*)/G protein-coupled estrogen receptor 1 (GPER), and HLG-A axes in breast, cervical, and ESCC cancers; HOTAIR/Mir206 interaction promotes tumor growth, migration, invasion, and EMT by induction of cyclin D genes, anti-apoptotic gene Bcl-w, and chemokine ligand CCL2 in breast, ovarian, and colon cancers and medulloblastoma.

HOTAIR/miRNAs interactions are able to interfere with different cellular processes during cancer development [29]. The analysis of the present data in the literature has shown that HOTAIR acts mainly as a sponge for different miRNAs, reducing their expression and causing the upregulation of a series of miRNA targets, including many oncogenes [108,179,180,181] (Figure 1).

For this reason, the possibility of intervening by perturbing this complex network of interactions could strongly suggest new therapeutic strategies.

Whereas miRNA-based therapeutics have recently shown to be feasible and safe in humans with encouraging results in early phase clinical trials [109,182,183], for lncRNAs, the situation is more complex, because of their ability to have different cellular localization.

New approaches should be optimized to interfere with HOTAIR/miRNAs complexes also by means of vector-based strategies, which, by silencing HOTAIR, could release miRNAs, restoring their ability to de-regulate target genes involved in tumor progression.

Although the possibility to translate these strategies into clinical practice is complicated, the discovery of new and complex relationships between miRNAs and lncRNAs is creating a new scenario for the prognostic therapeutic definition of cancer patients and for the optimization of new tools for cancer.

## Figures and Tables

**Table 1:** HOTAIR miRNA targets in human cancers.

miRNA ID	HOTAIR Role on Expression	Tumor Types	References
miR-1	suppression	Liver cancer, medulloblastoma	[103,104]
miR-7	suppression	Breast cancer	[105]
miR-17-5p	suppression	Cervical cancer	[106]
miR-34a	suppression	Breast, pancreatic, gastric, prostate, colon cancers	[101,102,107,108,109]
miR-101	suppression	Head and neck cancer	[110]
miR-101-3p	inverse suppression	Gastric cancer	[37]
miR-122	suppression	Liver cancer	[111]
miR-124	suppression	Gastric cancer	[108]
miR-130a-5p	suppression	esophageal squamous cell carcinoma, gall bladder	[112]
miR-138-5p	suppression	Ovarian cancer	[113]
miR-141	inverse suppression	Kidney cancer, glioma	[35,114]
miR-145	suppression	Liver cancer	[115]
miR-146a-5p	inverse suppression	Breast cancer	[34]
miR-148a	suppression	Breast, cervical, esophageal squamous cell cancers	[116,117]
miR-148b-3p	suppression	Glioma	[118]
miR-152	suppression	Prostate, gastric cancers	[119,120]
miR-193a	suppression	Prostate cancer, myeloid leukemia	[121,122]
miR-200c	suppression	Ovarian cancer	[123]
miR-204	suppression	Breast, kidney cancers	[124,125]
miR-205	suppression	Bladder cancer	[24]
miR-206	suppression	Breast, colon cancer, ovarian cancer, medulloblastoma	[22,23,104,126]
miR-214	suppression	Colon cancer	[127]
miR-217	suppression	Lung, kidney, gastric cancers, osteosarcoma	[128,129,130]
miR-218	suppression	Breast, liver cancers	[131,132]
miR-326	suppression	Lung cancer, colon cancer, glioma	[133,134,135,136]
miR-331-3p	suppression	Gastric cancer	[137]
miR-449b	suppression	Breast cancer	[138]
miR-454-3p	suppression	Gastric cancer, chondrosarcoma	[139,140]
miR-545	suppression	Colon cancer	[141]
miR-613	suppression	Pancreatic cancer, retinoblastoma	[142,143]
miR-663b	suppression	Pancreatic cancer	[144]

**Table 2:** Most used bioinformatic tools for miRNA/lncRNA sequence-based prediction.

Name	URL	Applications
Diana Tools	http://diana.imis.athena-innovation.gr/DianaTools/index.php	A package that provides algorithms, databases, and software for interpreting and archiving data in a systematic framework starting from deep sequencing data, the annotation of miRNA regulatory elements, and targets to define the role of ncRNAs in different diseases.
GDCRNATools	http://bioconductor.org/packages/devel/bioc/html/GDCRNATools.html	An R package that provides a standard tool to downloading, organizing, and integrative analyzing of RNA expression data in the Genomic Data Commons (GDC) portal, deciphering mainly the lncRNA-mRNA-related ceRNAs regulatory network in cancer.
Microcosm Targets	http://www.ebi.ac.uk/enright-srv/microcosm/htdocs/targets/v5/	A web resource containing computationally predicted targets for miRNAs across many species, including humans. MicroCosm Targets database uses dynamic programming alignment to identify highly complementary miRNA–mRNA pairs.
miRanda	http://www.mirbase.org/	The miRBase website provides a wide range of information on published miRNAs, including their sequences, their biogenesis precursors, genome coordinates and context, literature references, deep sequencing expression data, and community-driven annotation.
miRcode	http://www.mircode.org/	The miRcode web interface provides basic search functionality for finding putative miRNA–target sites in lncRNAs of interest or predicted targets of specific miRNAs.
MiRDB	http://www.mirdb.org/	An online database able to predict miRNAs target by a bioinformatics tool, MirTarget, which was developed by analyzing thousands of miRNA–target interactions from high-throughput sequencing experiments.
miRge	http://atlas.pathology.jhu.edu/baras/miRge.html	MiRge employs a Bayesian alignment approach, whereby reads are sequentially aligned against customized mature miRNA, hairpin miRNA, noncoding RNA, and mRNA sequence libraries. Reads for all other RNA species are provided, which is useful for identifying potential contaminants and optimizing small RNA purification strategies.
miRLAB	https://bioconductor.org/packages/release/bioc/html/miRLAB.html	An R package for automating the procedure of inferring and validating miRNA–mRNA regulatory relationships. It includes a pipeline to obtain matched miRNA and mRNA expression datasets directly from TCGA, the functions for validating the predictions using experimentally validated miRNA target data and miRNA perturbation data.
miRNApath	https://bioconductor.org/packages/release/bioc/html/miRNApath.html	A package for provision pathway enrichment techniques for miRNA expression data. miRNApath online database uses miRNA target genes to link miRNAs to metabolic pathways.
miRTarBase	http://mirtarbase.mbc.nctu.edu.tw/	A database containing miRNA–target interactions (MTIs). The collected MTIs are validated experimentally by reporter assays, Western blot, or microarray experiments with overexpression or knockdown of miRNAs.
PicTar	http://www.pictar.org/	A computational method for identifying common targets of miRNAs. Through statistical tests using genome-wide alignments of eight vertebrate genomes, PicTar is able to specifically recover published miRNA targets.
RegRNA	http://regrna.mbc.nctu.edu.tw/html/prediction.html	An integrated web server to identify homologs of the regulatory RNA motifs and elements against an input mRNA sequence. RegRNA displays prediction results in a graphical interface generated by various integrated analytical tools, and allows users to annotate their own experimental sequences or to discover homologs of their desired motifs.
RNA22	https://cm.jefferson.edu/rna22/	A pattern-based method for the identification of miRNA binding sites and their corresponding heteroduplexes.
starBase v2.0	http://starbase.sysu.edu.cn/	A database able to provide: the miRNA-pseudogene interaction networks, interaction maps between miRNAs and circRNAs, ceRNA functional networks based on miRNA–target interactions overlapping with high-throughput CLIP-Seq data, miRNA–lncRNA interactions, and a variety of interfaces and graphic visualizations to facilitate analysis of CLIP-Seq data, RBP binding sites, miRNA targets, and ceRNA regulatory networks in normal and cancer cells.
TargetScan	http://www.targetscan.org/	A web server that predicts miRNA target genes by searching for the presence of 6- to 8-mer sites that match the seed region of a given miRNA and make use of species alignment to locate conserved sites.
TargetScore	https://bioconductor.org/packages/release/bioc/html/TargetScore.html	A Bayesian probabilistic scoring method taking into account the fold-change due to miRNA overexpression and sequence-based information.
