# Correction: Cantile et al. Functional Interaction among lncRNA HOTAIR and MicroRNAs in Cancer and Other Human Diseases. *Cancers* 2021, *13*, 570

**DOI:** 10.3390/cancers17244015

**Published:** 2025-12-17

**Authors:** Monica Cantile, Maurizio Di Bonito, Maura Tracey De Bellis, Gerardo Botti

**Affiliations:** 1 Pathology Unit, Istituto Nazionale Tumori-Irccs-Fondazione G.Pascale, 80131 Naples, Italy; m.dibonito@istitutotumori.na.it; 2 Scientific Direction, Istituto Nazionale Tumori-Irccs-Fondazione G.Pascale, 80131 Naples, Italy; maura.traceydebellis@istitutotumori.na.it (M.T.D.B.); g.botti@istitutotumori.na.it (G.B.)

In the original publication [1], there were several references cited that have been retracted. Corrections have been made as follows:

Reference Updates:

The authors have deleted the following retracted references (previously cited as Refs. [55,62,86,98,107,122,135,138,146,150,187]). With this correction, the order of some references has been adjusted accordingly. The correction does not include referenced data, and the scientific conclusions have not been changed. This correction was approved by the Academic Editor. The original publication has also been updated.
